# Clustering Characterization of Acoustic Emission Signals Belonging to Twinning and Dislocation Slip during Plastic Deformation of Polycrystalline Sn

**DOI:** 10.3390/ma15196696

**Published:** 2022-09-27

**Authors:** László Z. Tóth, Lajos Daróczi, Tarek Y. Elrasasi, Dezső L. Beke

**Affiliations:** 1 Department of Solid State Physics, University of Debrecen, P.O. Box 400, H-4002 Debrecen, Hungary; 2 Department of Physics, Faculty of Science, Benha University, Benha 13518, Egypt

**Keywords:** clustering, acoustic emission, Sn, plasticity, twinning, dislocation slip

## Abstract

Results of acoustic emission (AE) measurements, carried out during plastic deformation of polycrystalline Sn samples, are analyzed by the adaptive sequential k-means method. The acoustic avalanches, originating from different sources, are separated on the basis of their spectral properties, that is, sorted into clusters, presented both on the so-called feature space (energy-median frequency plot) and on the power spectral density (PSD) curves. We found that one cluster in every measurement belongs to background vibrations, while the remaining ones are clearly attributed to twinning as well as dislocation slips at −30 °C and 25 °C, respectively. Interestingly, fingerprints of the well-known “ringing” of AE signals are present in different weights on the PSD curves. The energy and size distributions of the avalanches, corresponding to twinning and dislocation slips, show a bit different power-law exponents from those obtained earlier by fitting all AE signals without cluster separation. The maximum-likelihood estimation of the avalanche energy (ε) and size (τ) exponents provide ε=1.57±0.05 (at −30 °C) and ε=1.35±0.1 (at 25 °C), as well as τ=1.92±0.05 (at −30 °C) and τ= 1.55±0.1 (at 25 °C). The clustering analysis provides not only a manner to eliminate the background noise, but the characteristic avalanche shapes are also different for the two mechanisms, as it is visible on the PSD curves. Thus, we have illustrated that this clustering analysis is very useful in discriminating between different AE sources and can provide more realistic estimates, for example, for the characteristic exponents as compared to the classical hit-based approach where the exponents reflect an average value, containing hits from the low-frequency mechanical vibrations of the test machine, too.

## 1. Introduction

Acoustic emission (AE) is a non-destructive measurement method, which has high sensitivity, and thus, it is widely used for different scientific and engineering purposes [1,2,3,4,5,6,7,8,9,10,11]. The AE signals are fingerprints of intermittent microscopic processes accompanied by sudden local changes leading to the emission of acoustic signals [4,11,12,13,14,15,16]. The above citations also illustrate that the evaluation of the measured signals is continuously developing, since the information provided by AE or by nonlinear ultrasonic methods [16] is rich in different microscopic aspects of the investigated crackling noise (avalanches of AE signals). Thus, recent analyses go beyond the calculation of exponents (α) of various avalanche properties, describing the probability density of the given x parameter (x can be the amplitude (A), energy (E), size (S), duration (D), …):

(1) $$PDF\left(x\right)\propto x^{-\alpha }\exp-x/x_{C},$$

where $x_{C}$ is the cut-off value [17]. The focus is much more on AE waveforms [11,18], the avalanche shapes [9,11,19,20], or on the classification of the avalanches into so-called clusters [6,7,8] to separate the avalanches originating from different sources into groups, based on their properties. Using such a method, one can filter the accidentally detected false signals, or study the relationship of two or more physical phenomena, within one measurement [18].

It was shown recently [10] that by plotting the AE signals on an energy-amplitude plot, where each avalanche is represented by its energy and amplitude, we can observe multiple groups of avalanches, arranged along different lines if the noise spectra are heterogeneous. For more complex cases, we need an automated algorithm for classification [14,21,22]. The simplest way to classify any dataset is the classical k-means algorithm. For this, we have to choose two (or more) properties of the avalanches, for acoustic emission signals, usually one from each time domain, which can be the energy or the size of the avalanches,

(2) $$E=\frac{1}{R}\int_{start}^{finish}{\left(U\left(t\right)\right)}^{2}dt,$$

(3) $$S=\int_{start}^{finish}U\left(t\right)dt,$$

where R is an arbitrary resistance, and U(t) is the measured signal. The other one should be from the frequency domain, which is usually the median frequency, $f_{med}$ of the avalanche. It is determined from the power spectral density (PSD) function is given as:

(4) $$\int_{f_{min}}^{f_{med}}PSD\left(f\right)df=\int_{f_{med}}^{f_{max}}PSD\left(f\right)df.$$

Thereafter, the AE avalanches are plotted on the so-called feature space, as the function of the above-mentioned parameters. If the dataset contains members with significantly different properties, one can observe groups, that is, clusters on the feature space. The points of these clusters are distributed around a characteristic point, the centroid of the cluster, which is calculated as the average of the cluster members. The clustering process means to find the centroids of the clusters and assign every data point to one centroid. In the case of the classical method, this is an iterative process, where we choose random initial centroids, but we have to know the number of clusters a priori, and thus it is necessary to run the calculation multiple times, changing the number and the positions of the initial centroids.

To solve this problem, the method was improved. In the new algorithm [6], the number of clusters can change during the calculation, under the control of the coarsening and refinement parameters, to find the optimal number of clusters. This method can be used even during the measurement, when we do not know all AE avalanches yet, hence, it is called the adaptive sequential k-means (ASK) algorithm [6]. In the case of the ASK method, the feature space is used only for the demonstration of the results, the base of the clustering is the power spectral density function of the avalanche (calculated by fast Fourier transform), therefore, the centroids of the clusters are the average PSD functions of the cluster members. The PSD functions are discrete, they consist of n number of frequency components, thus we can consider the application of the PSD curve as an n-dimensional feature space.

The dominant deformation mechanism in a given material depends on various parameters, such as the rate of the deformation [23] and the temperature [24,25,26], or on the crystallographic direction of the deformation [27]. For tin, at low temperatures, the deformation takes place with twinning at low deformation rates, which turns into the collective motion of dislocations at higher temperatures [26]. Due to the intermittent character of the deformation process, we can detect acoustic emission signals. It was shown [26], that by changing the temperature, and consequently, changing the deformation mechanism, the characteristics of the detected acoustic emission signal also change. With increasing temperature, the number of avalanches, as well as a set of exponents of different avalanche parameters (energy, size) decreased significantly. However, as it is typical in other AE measurements too, the characteristic exponents, calculated on the basis of Equation (1), are obtained by taking into account all the detected AE signals. Thus, their values can be considered average values (reflecting also the contributions from different types of AE signals). For obtaining real exponents, belonging to the “pure” signals from twinning or dislocation slips, the separation of AE signals on the basis of their spectral processes is desired.

In this study, in order to study the AE signals in more detail, in accordance with the results of [26], we carry out acoustic emission measurement during the deformation of polycrystalline tin samples at two characteristic temperatures belonging to twinning as well as dislocation slip. The recorded signals will be processed with the means of the ASK method to explore their differences in the spectral properties, and to see what other noise sources are active besides the dominant deformation mechanisms. As a result, we will obtain new, corrected characteristic exponents belonging to real twining and dislocation slips, as compared to those obtained from using all AE signals. The importance of our approach, for the selection out of signals distorted by the so-called “ringing” of the sample-sensor system, can be emphasized by referring to one of the most important conclusions drawn in [12]: “the observed AE jerk profiles…says little about the local avalanche mechanism”. Thus, our separation of signals will lead to results reflecting only the properties of those, which belong to twinning or dislocation slips.

The method used in this article can be applied for the partition and classification of different local stress relaxation mechanisms producing AE signals, since they should have different waveforms and PSD functions, in general. Thus, in situ AE monitoring with adaptive sequential k-means (ASK) algorithm [6] helps to separate different local stress relaxation mechanisms during plastic deformations and other structural changes (e.g., during martensitic transformations) as it is illustrated in references [6,7,8,13,14,15,17,28,29].

## 2. Materials and Methods

Acoustic emission measurements were performed during compression tests at −30 °C and +25 °C. The details and the schematic of the experimental arrangement are shown in Ref. [26]. The components of the system (acoustic sensor, temperature regulation) were mounted on a Chatillon TCD 225 tensile test console producing a constant deformation rate $\dot{\varepsilon }=0.15\;s^{-1}$.

The original dimensions of the cylindrical tin samples were a 3-mm diameter and 3-mm height, which changed to 1 mm during the deformation. The acoustic signals were detected by a Micro-100S (Physical Acoustic Corporation) piezoelectric sensor connected by a long steel waveguide to the sample, to protect it from the widely variable temperatures (the temperature of the sample can be as low as −60 °C, or as high as 100 °C in other experiments [30,31]). The setup has a home-made 60 dB grounded base amplifier, with very good transmissibility in the 0 Hz–200 kHz frequency range. The signals were recorded using a National Instruments PCI-6111 multifunction data acquisition board, with a 5 MS/s/channel sampling rate.

The data analysis was performed offline. First of all, digital band-pass filtering was applied (10 kHz–1 MHz) to reduce the low-frequency oscillations of the baseline and the high-frequency background noise. The avalanches were identified with the classic threshold-based method, using a 40-mV threshold level and 80-µs hit detection time [32].

Thereafter, the avalanches obtained at different temperatures were sorted using the ASK algorithm [6] (see also the corresponding paragraphs and Equations (2)–(4) in the previous chapter). The base of the sorting was the power spectral density curve, which was calculated for every avalanche with Welch’s method. The difference ($D_{PQ}$) between two discrete PSD curves (P(f) and Q(f)) was calculated with the Symmetric Kullback–Leibler divergence:

(5) $$D_{PQ}=\underset{f\in \chi }{\sum }P\left(f\right)\log\frac{P\left(f\right)}{Q\left(f\right)}+\underset{f\in \chi }{\sum }Q\left(f\right)\log\frac{Q\left(f\right)}{P\left(f\right)}.$$

Here, χ is the probability space, where P(f) and Q(f) are defined (the set of the frequency components, determined by the fast Fourier transform). For more accurate results, the combination of the classic and adaptive sequential k-means algorithm was applied. The initial centroids were determined by the ASK method [6], but the final clusters were obtained with the classic, iterative method. After each iteration, the possibility of merging clusters was investigated, and during the iterations, a new cluster was defined, if an avalanche was processed with a much different PSD function, according to the rules of the ASK method. The iteration came to an end when nothing changed after the previous iteration.

## 3. Results

### 3.1. Low-Temperature Measurements

**Figure 1:**
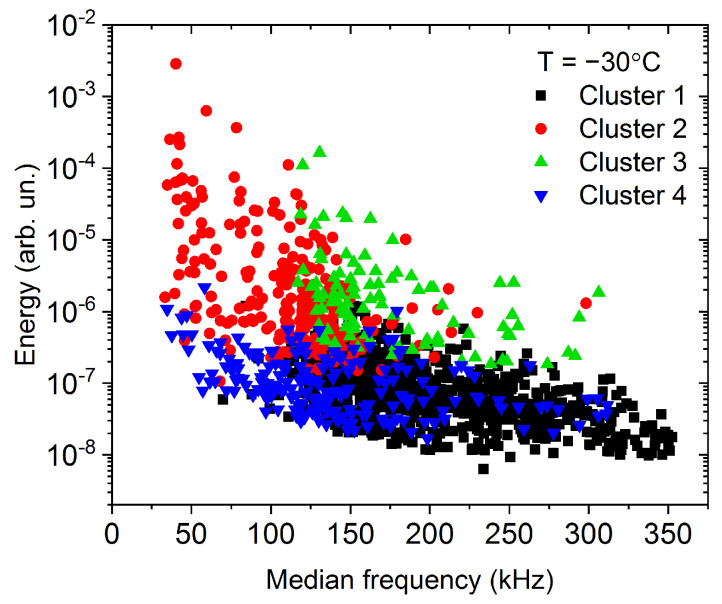
Energy—median frequency feature space for the measurement at −30 °C.

The 1476 AE avalanches, detected at −30 °C, were classified into four clusters. Figure 1 shows the results, where the clusters are represented on the energy vs. median frequency feature space.

**Figure 2:**
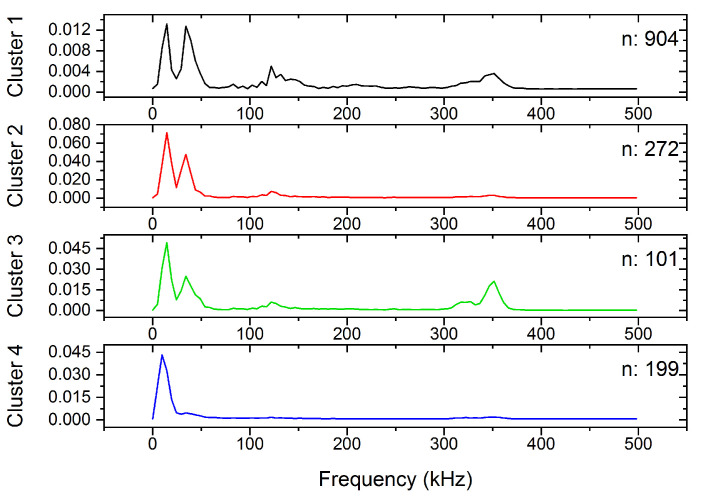
The average PSD functions, as the centroids of the clusters, including the number of avalanches at −30 °C.

**Figure 3:**
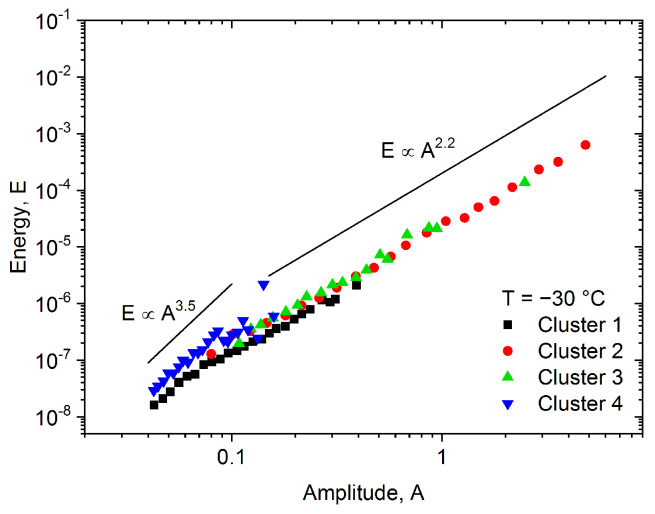
Energy vs. amplitude correlation for all clusters obtained at −30 °C. The exponent of the scaling relation is in the acceptable range only for Clusters 2 and 3 (and for a part of Cluster 1), while for Cluster 4 it is out of the predicted range (>3).

Although the clusters are not fully separated in Figure 1, (due to the overlap between the clusters), the avalanches belonging to different clusters are nicely grouped at certain regions of the feature space. Figure 2 shows the centroids of the clusters, that is, the average power spectral density functions. Additionally, Figure 3 shows the energy–amplitude correlation [33] (see also the Discussions).

### 3.2. Room Temperature Measurements

**Figure 4:**
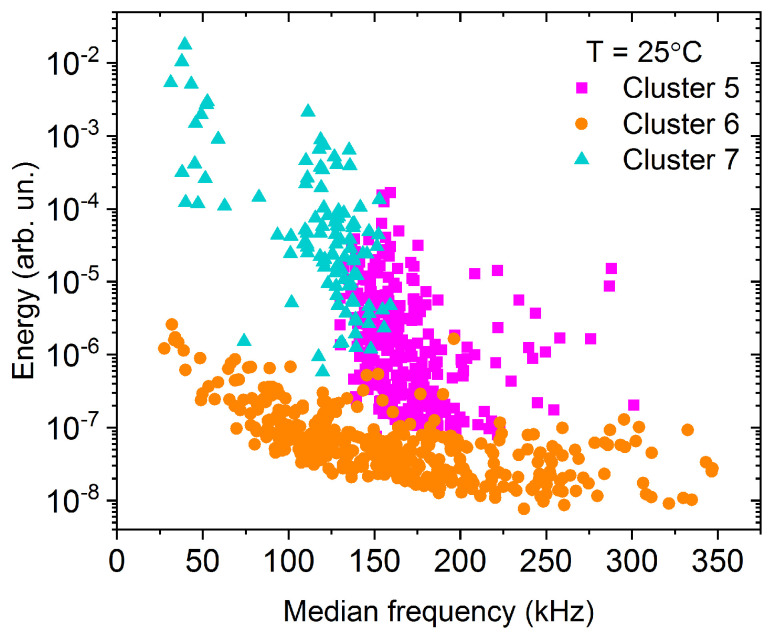
Energy—median frequency feature space for the measurement at 25 °C.

**Figure 5:**
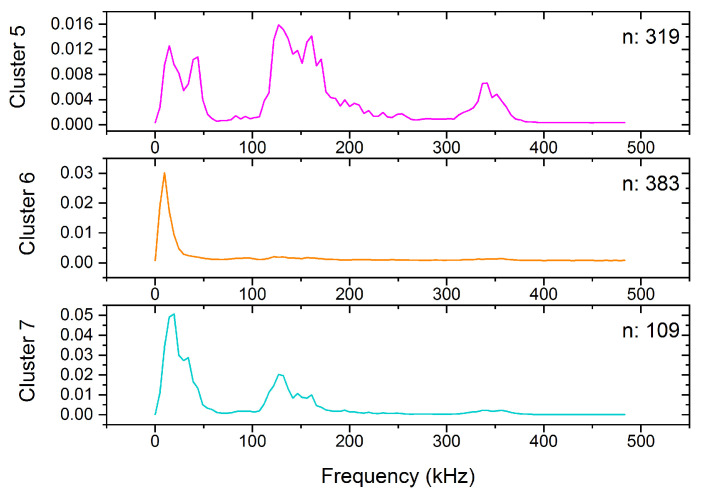
The average PSD functions, as the centroids of the clusters, including the number of avalanches at 25 °C.

**Figure 6:**
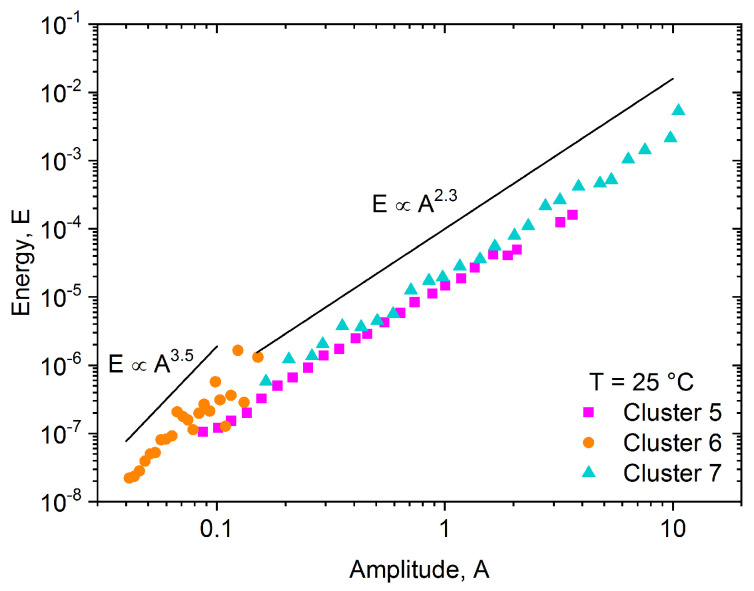
Energy vs. amplitude function for all clusters obtained at 25 °C. The exponent of the scaling relation is in the acceptable range only for Clusters 5 and 7.

Two sets of measurements were done at room temperature, which can be high enough that the deformation mechanism of tin is different but we still have a satisfying number of acoustic avalanches [26]. The results, similar to the results obtained at −30 °C, are shown on the feature space (Figure 4), in the power spectral density functions (Figure 5) and in the energy-amplitude correlation (Figure 6).

## 4. Discussion

### 4.1. Low-Temperature Measurements

From Figure 2, as well as from Figure 3, showing the energy–amplitude correlation [33], one can see the similarities and differences between the clusters, and we can make assumptions regarding the origin of the acoustic emission avalanches.

Cluster 4, has only low-frequency components, and it contains low-energy acoustic hits. The slope of the energy–amplitude plot is fairly different than the slope corresponding to other clusters (particularly Clusters 2 and 3). Thus, we can conclude, that these acoustic hits plausibly correspond to low-frequency mechanical vibrations, originating from the tensile test machine.

**Figure 7:**
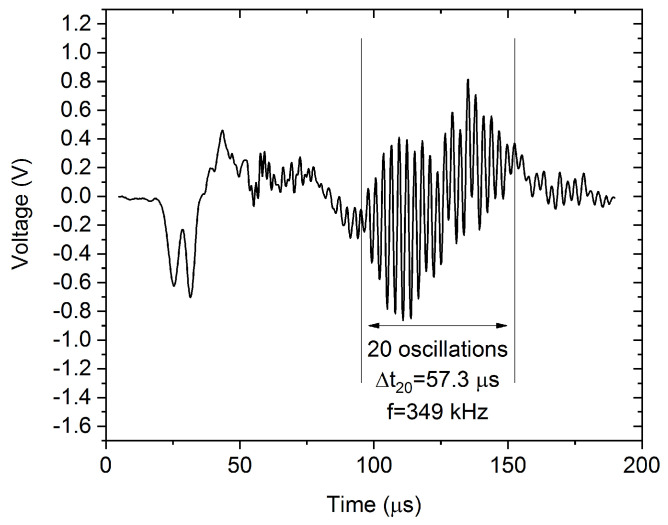
Characteristic avalanche of Cluster 3. The ringing is the most spectacular at the selected part of the avalanches, resulting in ringing of 349 kHz frequency, as was expected from the PSD curve of Cluster 3.

Clusters 2 and 3 are very similar, the only difference is that Cluster 3 has high-frequency components around 350 kHz, while Cluster 2 has negligible contribution in this range. This behavior is related to the transfer function of the sample-sensor system: one can observe the so-called ringing effect on a certain part, or on the whole avalanche, when the transfer function has a high influence on the detected signal [12,34]. For longer avalanches, the effect of ringing on the slope of the logE versus logA plot is usually negligible [34]. Thus, Clusters 2 and 3 probably have the same origin, the only difference is the duration of the avalanches. This is confirmed by Figure 3, where the slopes corresponding to the two clusters are the same within the experimental error, but Cluster 2 contains avalanches with higher amplitude and energy (and plausibly longer duration). Figure 7 shows a characteristic AE avalanche from Cluster 3, where the fingerprint of the ringing effect is visible. The ringing is the most spectacular in the selected section of the avalanche. This section contains 20 oscillations and its duration is about 57.3 µs, corresponding to 349 kHz frequency, which is in good agreement with the peak on the PSD curve of Cluster 3.

The number of avalanches in Cluster 1 is very high compared to the others, but the energy and amplitude values of these avalanches are low and the deviation of points on the first part of the plot in Figure 3 is most probably due to the threshold effects and distortion effects of ringing [12,34]. Thus, this cluster can also be identified as belonging to the real deformation mechanism (similar to Clusters 2 and 3).

Recently, it turned out [34] that in the theoretically predicted averaged temporal shape of avalanches at a fixed area, $U\left(t\right)=atexp^{-bt}$, a and b are not universal constants [35,36], rather they can be given as $b=1/2R^{2}$ and $a\propto A^{\varphi }\propto A/R$, where R is the rising time and φ is a mechanism-dependent constant, 0≤φ≤1. It was shown in Ref. [34], that it appears also in the scaling relations $E\propto A^{3-\varphi }$ and $S\propto A^{2-\varphi }$. We can observe in Figure 3, that the exponent of the power law relation between the energy and the amplitude has the value of 2.2 for Clusters 2 and 3 (and for Cluster 1 at higher amplitudes), which is in the acceptable range, proposed by Ref. [34]. At the same time, the exponent of the energy–amplitude scaling relation for Cluster 4 is out of the acceptable range (3.5). This confirms the above hypothesis that Cluster 4 may belong to background vibrations while the other clusters probably contain useful acoustic emission avalanches, originating from the deformation mechanism (twinning) of the sample.

It is worth mentioning, that we also carried out several checking runs at 0 °C and similar clusters with similar properties were obtained.

### 4.2. Room-Temperature Measurements

The cluster analysis of the 811 acoustic emission avalanches detected during the room temperature measurement resulted in three clusters (named Cluster 5–7, to avoid mixing up with the clusters of the low-temperature measurement). Starting with Cluster 6, we can see that based on the position of the points on the feature space (Figure 4), on the shape of the PSD curve (Figure 5), and also based on the energy-amplitude correlation (Figure 6), this cluster is identical with Cluster 4 obtained at −30 °C, and thus it is plausibly attributed to background vibrations.

In contrast to Cluster 6, Cluster 5 of the room-temperature measurement was not observed at −30 °C. It is significantly different from any other clusters due to the high contribution around 150 kHz on the PSD curve shown in Figure 5. Thus, we can conclude, that this corresponds to a different source of acoustic emission signals (dislocation slips) as those belonging to Clusters 2 and 3 at −30 °C (twinning). The peak of the ringing contribution (at about 350 kHz) is also present on the PSD curve (like for Clusters 1 and 3) since the amplitudes (and consequently the duration times) of these avalanches are starting from very small values, where the transfer effects can be remarkable. On the other hand, while the PSD curve of Cluster 7 shows also a significant contribution near 150 kHz, there is only a very small peak at about 35 kHz. The slopes of the energy–amplitude scaling at room temperature (Figure 6) behave similarly for Clusters 5 and 7.

**Figure 8:**
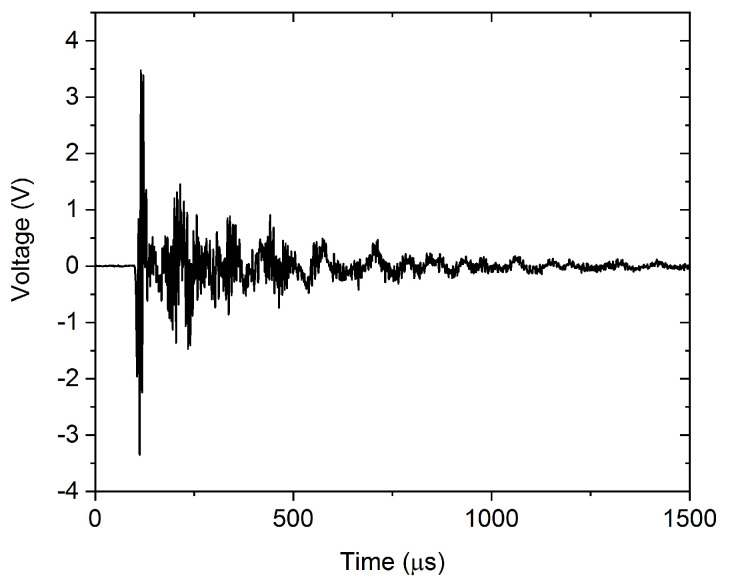
Characteristic avalanche of Cluster 5 at room temperature. The high-frequency ringing is visible, together with slower oscillations around 150 kHz, predicted by the PSD curve of Cluster 5 in Figure 5.

Figure 8 shows a typical AE avalanche belonging to Cluster 5. However, both the amplitude and duration of the avalanche are considerably higher, than for the one shown in Figure 4, the high-frequency ringing is still present. In addition to the high-frequency ringing, we can observe a slower, collective oscillation of the signal, corresponding to about 150 kHz, as we can expect from the PSD curve of Cluster 5 in Figure 5. Comparing Figure 7 and Figure 8, we can conclude that the typical AE waveform is considerably different for twinning and dislocation slip, in accordance with the PSD functions, shown in Figure 2 and Figure 5.

### 4.3. Statistical Analysis of the Clusters

We carried out the usual statistical analyses [26] separately for the different clusters at both temperatures to obtain the characteristic exponents according to Equation (1). After the avalanches of each measurement were detected and classified, the probability density functions of avalanche energy and size were determined with the means of logarithmic binning to make the first estimation for the exponent values.

**Figure 9:**
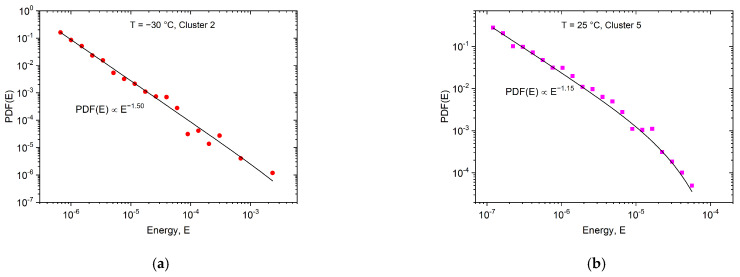
Probability density functions of the avalanche energies for Cluster 2 (**a**) and for Cluster 5 (**b**).

**Figure 10:**
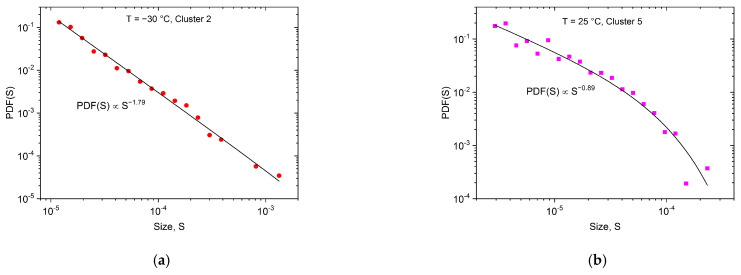
Probability density functions of the avalanche sizes for Cluster 2 (**a**) and for Cluster 5 (**b**).

According to the results of the clustering process, Clusters 2 and 3 contain acoustic emission avalanches, originating from twinning at −30 °C, while at room temperature Clusters 5 and 7 belong to dislocation slips [18,26]. Figure 9 and Figure 10 show, as an illustration, the probability density functions of the avalanche energies and sizes for Clusters 2 and 5 only, because of the satisfying number of avalanches.

It can be seen from the values of the exponents given in Figure 9 and Figure 10, that indeed different exponents belong to the two deformation processes, as is expected on the basis of the results of Ref. [26].

**Figure 11:**
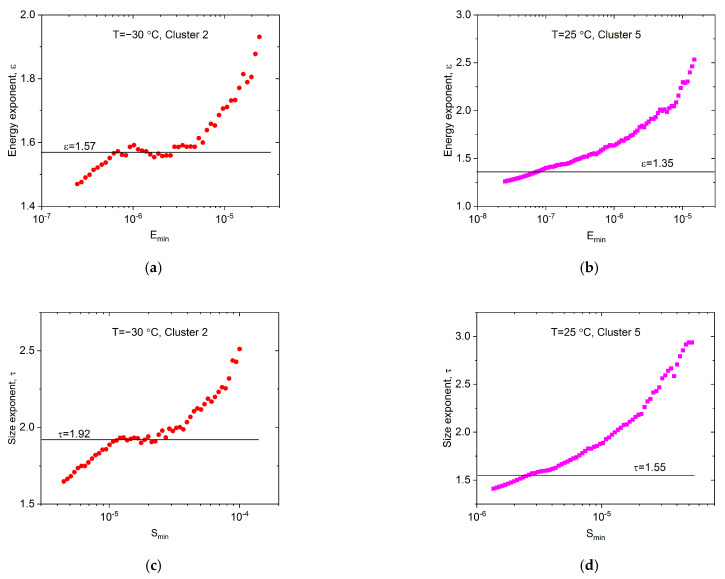
Maximum-Likelihood estimations for the energy (**a**,**b**) and size (**c**,**d**) exponents at −30 °C (**a**,**c**) as well as 25 °C (**b**,**d**). For −30 °C the corresponding exponents are given by the plateaus, while for 25 °C the exponents are approximated because of the exponential damping of the probability density functions.

However, there are two aspects that allude to caution regarding the above results: there exists a minimum mean-field theoretical value for the energy exponent, ε≥1.33 [17,37,38], as well as the well-known scaling rule (τ−1)/(ε−1)=1.5 [17]. Both seem to be violated for the high-temperature values. The same is valid for the exponents obtained in [26]: ε *=* 1.45 ± 0.05 (−60 °C) and ε = 1.20 ± 0.15 (50 °C), as well as the exponent of the avalanche size (τ) changed from τ *=* 1.9 ± 0.1 to τ= 1.0 ± 0.3 (from −60 °C to 50 °C, respectively), *where all AE hits were used for the evaluation*. This can be due to the relatively low number of AE avalanches in each cluster, as a result of which, the logarithmic boxing may have distortions as well as strong cut-off effects in Figure 9b and Figure 10b plots. Thus, we have to determine the exponents more accurately, independently of the logarithmic boxing, using the maximum-likelihood (ML) method [39,40] (the ML analysis in [26] was demonstrated for −30 °C only). Figure 11a–d shows the maximum-likelihood estimation for the energy and area belonging to Cluster 2 and Cluster 5, respectively.

First, the differences between Clusters 2 and 5 are clearly visible. The ML curves in Figure 11a,c *for the low-temperature measurements* (Cluster 2) have well-expressed plateaus, in accordance with the fact that the corresponding probability density functions are straight. Thus, the accurate exponent values are given by the plateaus: ε=1.57±0.05 and τ=1.92±0.05 for −30 °C. These values are close to the values of the fitting of the PDF and to the average values of Ref. [26]. *For the room temperature measurements*, the estimation of the exponent is not so straightforward due also to the presence of the significant cut-off effect on the PDF curves (Figure 9b and Figure 10b). For exponentially damped power-law distributions, there is a kink in the curve near the minimum value in the dataset, and the kink height overestimates the exponent value [40]. In this case, we can apply the approximation of Ref. [40], or extrapolate the ML curve toward the lower values, to find the exponent value that is asymptotically reached by the extrapolated curve. The room temperature exponents from this estimation are ε=1.35±0.1 and τ=1.55±0.1 for Cluster 5 (Figure 11b,d). These values are significantly higher than the exponent values determined from the logarithmically binned PDF functions, and they are also higher than the average values in Ref. [26]. Furthermore, these exponents satisfy the criteria for the minimum value, and the well-known scaling laws [17]. Moreover, the tendency of the exponents for the temperature change, similar to Ref. [26], is still observable. These exponent values, together with the exponents for all other clusters, are summarized in Table 1.

It can be seen in Table 1, that AE measurement can contain different contributions of acoustic emission sources, producing signals with considerably different statistical properties. In most of the cases, only some of these contributions carry useful information about the physical process, the others may be background noise, or simply distorted signals, which should be excluded from the evaluation. Inquiring into the data in Table 1, we can see that without clustering, considering every avalanche, we would get averaged probability density functions, and thus, inaccurate exponents

## 5. Conclusions

In order to carry out clustering characterization of AE signals of different origins, on the basis of the results of [26], uniaxial compression measurements were carried out on polycrystalline tin samples at two different temperatures, at −30 °C and room temperature, corresponding to twinning and dislocation slips, respectively. The recorded acoustic emission signals were processed with the means of an adaptive sequential k-means algorithm. The classification resulted in different clusters, assigned to background noise as well as to distorted and pure signals, originating from the dominant deformation mechanism at each temperature. Our results are in line with the results of Ref. [14], obtained from AE measurements in Mg alloys, supporting one of the main conclusions of this paper: “the ASK analysis proved to be a useful tool in discriminating between different sources of AE even if the classical hit-based approach reaches its limitations”.

Analyzing the statistical properties of the clusters, the main deformation mechanism at low temperatures was identified as Cluster 2, belonging to twinning. The corresponding energy and size exponents (ε *=* 1.57 ± 0.05 and τ *=* 1.92 ± 0.05, respectively) are in good agreement with the results of [26], indicating that in the averaged AE signals, this cluster (and Cluster 3; see also Table 1) has determining weight.

In the case of the room-temperature measurement, Clusters 5 and 7 correspond to the collective motion of dislocations. The energy and size exponents, determined also with the ML method, (ε *=* 1.35 ± 0.1 and τ *=* 1.55 ± 0.1) are larger than those obtained in [26] by using all measured AE signals. These new values are also in accordance with the expected minimal value of the energy exponent and the well-known scaling relation: they are smaller for dislocation slips than for twinning.

Thus, clustering characterization provided not only a manner to eliminate the background noise and noise from the low-frequency mechanical vibrations of the test machine but also confirmed that the characteristic avalanche shapes are also different for the two mechanisms, as is also visible on the PSD curves. Furthermore, the detailed statistical analysis resulted in more reliable exponent values. In addition, interestingly it was observed that fingerprints of the well-known “ringing” of AE signals are present in different weights on the PSD curves.

## Figures and Tables

**Table 1:** Energy and size exponent values for all clusters, determined with maximum-likelihood estimation. The values for Clusters 2 and 5, corresponding to twinning and dislocation slip are highlighted in bold, while the asterisk draws attention to the high uncertainty, caused by the extremely low number of avalanches (≈100) and/or by the relatively high number of small avalanches (Cluster 1). Clusters 4 and 6 are attributed to background vibrations (see also the text).

Temperature	Cluster (Number of Avalanches)	Energy Exponent, ε	Size Exponent, τ
−30 °C	1 (904)	1.3 ± 0.1 *	1.5 ± 0.1 *
	**2** (**272**)	**1.57 ± 0.05**	**1.92 ± 0.05**
	3 (101)	1.4 ± 0.2 *	1.55 ± 0.2 *
	4 (199)	1.6 ± 0.1	1.65 ± 0.1
25 °C	**5** (**319**)	**1.35 ± 0.1**	**1.55 ± 0.1**
	6 (383)	1.5 ± 0.1	1.6 ± 0.1
	7 (109)	1.35 ± 0.2 *	1.6 ± 0.2 *

## Data Availability

The data presented in this study are available on request from the corresponding author.
